# The role of microglia in mediating the effect of the environment in brain plasticity and behavior

**DOI:** 10.3389/fncel.2014.00390

**Published:** 2014-11-18

**Authors:** Igor Branchi, Silvia Alboni, Laura Maggi

**Affiliations:** ^1^Department of Cell Biology and Neuroscience, Istituto Superiore di SanitàRome, Italy; ^2^Department of Life Sciences, University of Modena and Reggio EmiliaModena, Italy; ^3^Department of Physiology and Pharmacology, Sapienza UniversityRome, Italy

**Keywords:** microglia, plasticity, environment, depression, behavior, stress

Major Depression (MD) affects 10–15% of the population worldwide and constitutes an enormous medical, societal, and economic burden. One of the prominent causes of such burden is the very limited understanding of the mechanisms underlying the psychopathology (Belmaker and Agam, 2008). Among the most relevant factors determining the onset of MD is the quality of the living environment, such as exposure to stressful life events (Cohen et al., 2007; Davidson and McEwen, 2012). In addition, increased inflammation, associated to conditions such as infection and immunotherapy, is reported to increase the likelihood to show depressive symptomatology (Dantzer et al., 2008; Han and Yu, 2014).

The nervous and the immune systems are engaged in an intense bidirectional interplay, along the ongoing changes in the living environment (Yirmiya and Goshen, 2011; McCusker and Kelley, 2013). In this perspective, microglia, the resident immune cells of the brain, have recently attracted a lot of interest in the biological psychiatry field (Muller, 2014). These cells can modify their features and function according to the inputs from the environment. Indeed, equipped with receptors for a plethora of molecules microglia cells can sense environmental changes over a time scale of minutes and respond performing diverse functions which, accordingly to the context, might result as either beneficial or harmful (Kierdorf and Prinz, 2013; Siskova and Tremblay, 2013), though factors regulating microglial transition across different functional states are not well-defined. For instance, these cells respond to sensory and behavioral experience (e.g., deprivation of visual stimuli, environmental enrichment) by modulating their interactions with neuronal circuits, notably regulating processes such as adult hippocampal neurogenesis (Ekdahl, 2012; Reshef et al., 2014) and elimination and formation of synapses (Paolicelli et al., 2011; Tremblay et al., 2011; Parkhurst et al., 2013; Sierra et al., 2014).

The role of microglia in interfacing environmental stimuli and changes in brain function has suggested that these cells may underlay the interplay between environmental stimuli and vulnerability to MD. In this context, the preclinical study performed by the group headed by Raz Yirmiya provides an interesting demonstration of the role played by the microglial cells in mediating the effects of stress on depression. Authors showed that stress exposure produced dynamic bi-directional alterations in microglia status that, in turn, are causally involved in stress-induced depressive-like behavior in rodents. They explored the effects of stress in three brain regions: the hippocampal dentate gyrus, the medial prefrontal cortex and the somatosensory cortex. They found that mice and rats exposed to unpredictable stress (US) show microglia modifications, mainly in the dentate gyrus of the hippocampal system, which are dependent on the duration of the US. Indeed, they demonstrated that a short-term exposure (1–2 days) to US results in a transient induction of microglia proliferation and activation (i.e., microglial cells assume an activated morphology and increased expression of activation markers) via IL-1\ IL-1R mediated signaling. However, following 3–4 days of US, a microglia decline was observed in stressed animals compared to controls as indicated by high levels of caspase-3 (a protease that mediates the execution-phase of apoptosis) and DNA fragmentation (a key feature of apoptosis). Moreover, they found that chronic (5 weeks) US exposure induced depressive-like behavior (namely, decreased sucrose preference and social exploration) associated with a lowered number of microglia cells, reduction in length of microglial processes and soma area, and decreased expression of microglial markers (i.e., Iba-1 and CD11b). In addition, Yirmiya and collaborators showed, through pharmacological manipulations, that counteracting the bi-directional changes in microglial activity induced by stress can prevent or reverse the depressive-like phenotype. In particular, the block of microglial activation during the early phase of stress exposure through administration of minocycline (a commonly used drug to inhibit microglial activation) or imipramine (an antidepressant with anti-inflammatory properties; Alboni et al., 2013) prevents, at long-term, specific stress-induced effects on microglia and behavior. Whereas, during the late phase of stress exposure, when microglial decline occurs, the stimulation of microglia through administration of lipopolysaccharide, macrophage colony-stimulating factor or granulocytes-macrophage colony-stimulating factor reverses the stress-induced depressive-like behaviors. These observations support a causal role for the dynamic microglial changes in triggering and maintaining depressive-like symptoms under stress conditions and suggest an inverted U-shaped relationship between time-dependent microglial status and behavioral outcome (Goshen et al., 2007; Kreisel et al., 2014).

Overall, the results of this paper open a new perspective about the role of microglia in the interplay between the quality of the environment and vulnerability to MD. We strengthen this finding, stressing the idea that microglia plays a key role in such interplay not only actively participating but being integral part of brain plasticity (Figure 1). Therefore, along with neuronal plasticity, microglial plasticity should be considered as a key component of the brain plasticity processes. In this perspective, neuronal plasticity, and brain plasticity are not synonyms, but the latter emerges from the plastic capabilities of both neurons and microglia, and glia in general, and from their interaction. Accordingly, it is warranted to explore brain plasticity in a comprehensive fashion, assessing neuronal plasticity (e.g., synaptic strength, spine, and dendritic modifications) in parallel with the dynamic changes in microglial status (e.g., morphological changes, phagocytic activity). This approach may lead to unravel novel molecular and cellular mechanisms underlying the onset and progression of psychopathologies to be exploited as targets for innovative therapeutic strategies.

**Figure 1:**
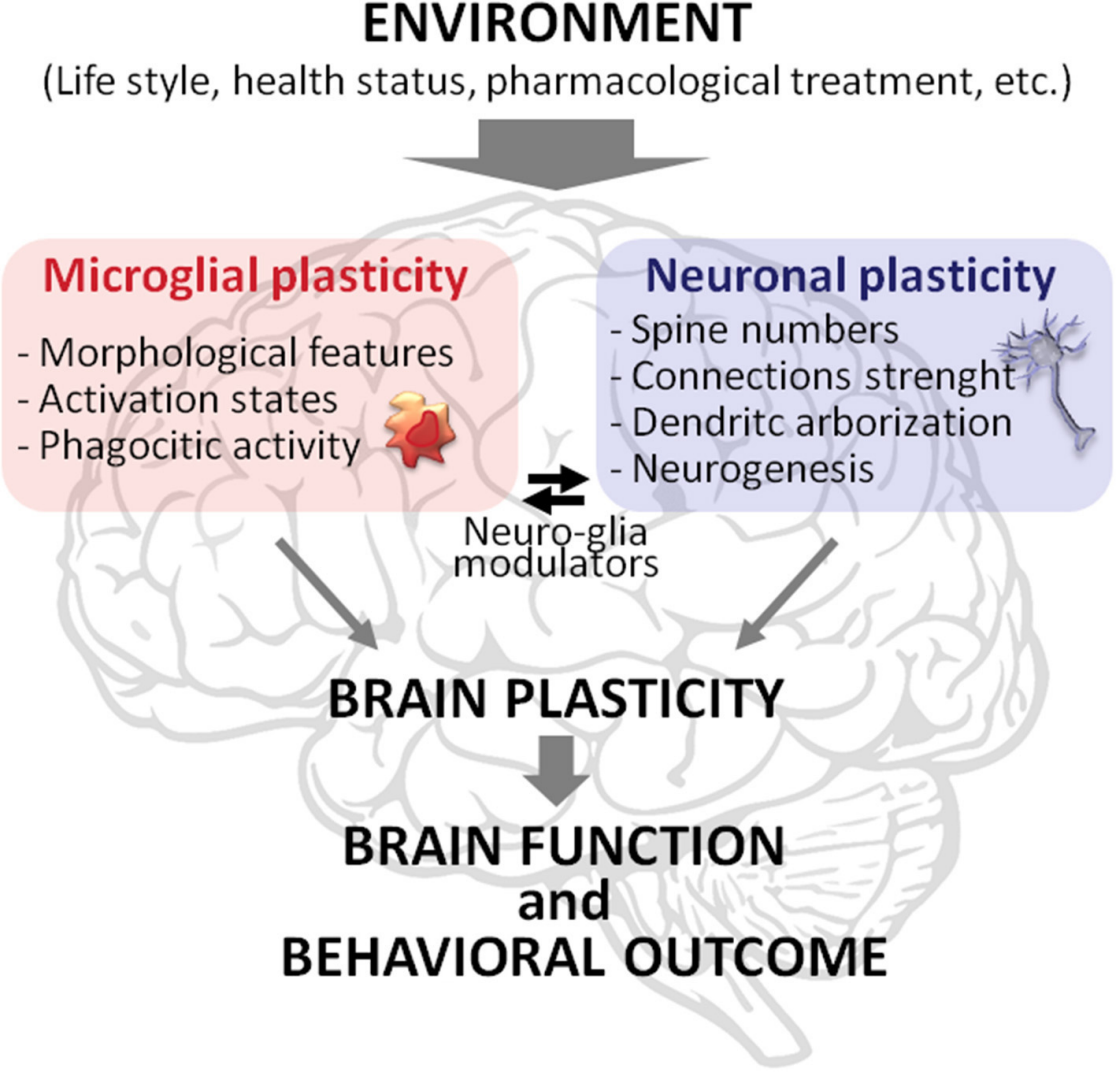
**Schematic representation of brain plasticity as a complex phenomenon emerging from the plastic capabilities of microglia and neurons and their interaction**. Microglia and neurons modify their morphology and functionality in response to environmental stimuli, such as exercise, diet, social interactions, health conditions, and pharmacological treatments. The modifications in these two cell types are intertwined. For instance, microglial phagocytic activity strongly affects neuronal spine remodeling while compounds released by neurons influence microglia status. These processes allow brain function and behavioral outcome to be tuned to the features of the environment.

## Conflict of interest statement

The authors declare that the research was conducted in the absence of any commercial or financial relationships that could be construed as a potential conflict of interest.
